# Evolutionary trends in the prevalence of anti-HDV antibodies among patients positive for HBsAg referred to a national laboratory in Cameroon from 2012 to 2017

**DOI:** 10.1186/s13104-019-4460-4

**Published:** 2019-07-15

**Authors:** Laurence Noubissi-Jouegouo, Marie Amougou Atsama, Paul Alain Tagnouokam-Ngoupo, Chavely Gwladys Monamele, Laure Ngono, Richard Njouom

**Affiliations:** 1 Virology Department, Centre Pasteur of Cameroon, Po Box 1274, Yaoundé, Cameroon; 2 School of Health Sciences, Catholic University of Central Africa, Yaoundé, Cameroon

**Keywords:** Trends, Prevalence, Anti-HDV antibodies, HBsAg, Cameroon

## Abstract

**Objective:**

The aim of this study was to update the data on the prevalence of anti-HDV antibodies in Cameroon.

**Results:**

Antibodies against hepatitis Delta virus (Anti-HDV) were found in 16.48% (95% CI 11.46–18.77%) of 426 hepatitis B virus surface antigen positive patients in Cameroon. Remarkably, they were significantly higher among people over 40 years and those living in the East and South regions of Cameroon at 66.7%, 50%, and 40%, respectively. These results suggest that older age and living in areas in the dense forest may be risk factors for Hepatitis D infection.

## Introduction

Hepatitis D virus (HDV), the causative agent of hepatitis delta is a satellite virus that uses the Hepatitis B virus (HBV) envelope for survival. HDV is a negative stranded RNA virus of 1.7 kb in size and the only representative of the Deltaviridae family [1, 2].Of the 250 million chronic HBV surface antigen (HBsAg) carriers worldwide, about 15–20 million are co-infected with HDV and the prevalence varies widely across regions [3]. HDV is considered to be the most severe form of viral hepatitis and transmission may occur via the parental route through contact with infectious body fluids, via sexual transmission as well as intrafamilial spread [4]. A high prevalence of anti-HDV has been described in HBsAg-positive patients in the Mediterranean region, the Middle East, Central Africa, the northern part of South America, Russia and Eastern Europe [5]. A recent meta-analysis on the prevalence of Hepatitis delta in sub-saharan Africa has shown that Central Africa is the most affected region with an overall HDV seroprevalence of 25.6% [6]. In Cameroon, a country located in Central Africa the first studies available two decades ago reported prevalences of 27.3% [7] among five different populations including pregnant women, prostitutes, transfused sickle cell patients, medical students, and people suffering from jaundice in Yaoundé (1991); and 6.5% [8] among healthy individuals in Nkongsamba (1986). In 2009, a study from two health facilities in Yaoundé reported a prevalence of 17.6% in HBsAg-positive subjects [9]. The most recent data obtained from the Demographic Health Survey (DHS) in 2011 showed a national prevalence of anti-HDV antibodies of 13.8% [10]. This study was then conducted to update the data on the prevalence of HDV in Cameroon in order to have more current data for health strategies implementation.

## Main text

### Methods

The Centre Pasteur of Cameroon (CPC) is one of the reference laboratories in Cameroon and plays a central role in Hepatitis country program. Data presented here were collected in the framework of routine activities of HDV diagnostic and no additional test was performed. Data were anonymously analyzed and reported in respect to patient confidentiality. Therefore, ethical clearance was not required for the study.

We conducted a cross-sectional study from January 2012 to December 2017 in 426 chronic HBsAg carriers sent by their physicians at CPC for the diagnosis of HDV infection. In Cameroon, HDV screening is demanded to HBsAg carriers as part of their initial medical follow-up tests in accordance with recommendations from the Cameroonian Society of Gastroenterology considering the endemicity of this infection in Central Africa. Plasma samples from HBsAg carriers were screened for HDV antibodies using a competitive ELISA assay, HDV Ab (Dia.Pro Diagnostic Bioprobes.S) with sensitivity and specificity of ≥ 98%. Negative and positive controls provided with the kit were run in each ELISA assay. The coloration intensity obtained was inversely proportional to the amount of antibodies against HDV present in the sample. Samples with a ratio (Threshold/OD of the sample) > 1.1 were considered seropositive for anti-HDV antibodies. Anti-HDV results from past HBsAg carriers during the period of 2012–2016 were retrieved from the CPC database. HDV antibodies within this period were as well screened using the HDV Ab ELISA assay (Dia.Pro Diagnostic Bioprobes.S). Socio-demographic data (age, gender and area of residence) were also recorded. Regarding statistical analysis, Logistic regression was used to evaluate risk factors. The male gender, individuals below 12 years, the center region and the year 2012 were used as reference to each group and the *T* test was used for the comparison of means. Chi- squared test was used to calculate the p-value (p^b^) for each group and the significance level was set at p < 0.05 and the confidence interval was set at 95%.

### Results

**Fig. 1:**
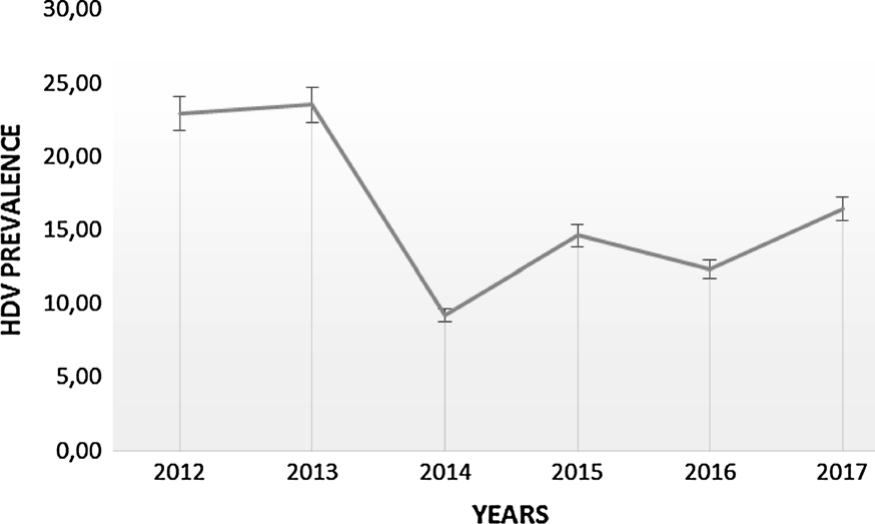
Temporal trend of HDV infection over time

The mean age of the study population was 28.7 ± 12.1 years [range 6–83 years] with 43% females. The overall prevalence of anti-HDV antibodies was 16.5% (95% CI 11.8–18.8%) ranging from 22.9% in 2012 to 16.4% in 2017 with the minimum in 2014 (9.2%) and the maximum in 2013 (23.5%). Table 1 shows the trends of anti-HDV prevalence over time and associated risk factors including gender, sex, and residence. The anti-HDV prevalence’s per year of sampling from 2012 to 2017 were respectively of 22.9%, 23.5%, 9.2%, 14.6%, 12.3%, and 16.4% (Fig. 1). People coming from 9 out of the 10 administrative regions of Cameroon participated in the study, among which only the Center, East, Littoral, North West, and South regions had people infected by HDV with prevalence of 14.3%, 40%, 40%, 50%, and 50%, respectively. However there were no samples to test from the Adamawa, North, West and South West regions. There were no statistically significant difference in anti-HDV prevalence according to gender and year of sampling. However, people over 40 years old and those residing in the East and South regions had significantly high prevalence of anti-HDV antibodies. These significantly high prevalences were as well noted within some specific years. Individuals over 40 years were the most at risk of HDV infection in the years 2012 and 2015 with OR = 3.71 and OR = 6.25, respectively. In 2014, the South region had an HDV-seroprevalence of 66.7% and at risk of infection with an OR of 27.20. A similarly high seroprevalence was noted in 2016 with the East region (OR = 8.167).

**Table 1:** Evolutionary trends in the prevalence of anti-HDV antibodies and associated risk factors

Years	Variable^a^	Anti-VHD +/N^b^	Prevalence % (95% CI)	p^c^	Crude OR (95% CI)	p-value
2012–2017	Gender			0.132		
	Female	22/183	12 (7.6–17.6)		1 (reference)	
	Male	42/243	17.3 (12.7–22.6)		0.654 (0.134–1.14)	0.134
	Age groups (years)			0.204		
	< 12	1/11	9.1 (0.22–41.3)		1 (reference)	
	[12–20]	18/108	16.7 (10.2–25.1)		0.814 (0.461–1.439)	0.480
	[21–40]	41/301	13.6 (9.9–18.0)		0.690 (0.394–1.208)	0.218
	> 40	4/6	66.7 (22.3–95.7)		8.852 (1.449–54.074)	0.005
	Regions			0.011		
	Center	54/377	14.3 (10.9–18.3)		1 (reference)	
	East	4/10	40 (12.2–73.8)		3.956 (1.084–14.43)	0.037
	Littoral	2/5	40 (5.2–85.3)		0.259 (0.042–1.582)	0.143
	North West	1/2	50 (1.2–98.7)		0.175 (0.011–2.826)	0.219
	South	3/6	50 (11.8–88.2)		5.885 (1.161–29.83)	0.032
	Adamawa	0/1	0	–	–	–
	North	0/2	0	–	–	–
	West	0/20	0	–	–	–
	South West	0/3	0	–	–	–
	Years			0.195		
	2012	8/35	22.9 (10.4–40.1)		1 (reference)	
	2013	12/51	23.5 (12.8–37.5)		0.523 (0.257–1.064)	0.074
	2014	7/76	9.2 (3.7–18.1)		1.918 (0.838–4.387)	0.123
	2015	13/89	14.6 (8.0–23.7)		1.042 (0.539–2.016)	0.902
	2016	14/114	12.3 (6.8–19.7)		1.363 (0.722–2.574)	0.340
	2017	10/61	16.4 (8.2–28.1)		0.886 (0.424–1.850)	0.746
2012	Gender			0.286		
	Female	4/12	33.3 (9.9–65.11)		1 (reference)	
	Male	4/23	17.4 (4.9–38.78)		2.375 (0.473–11.92)	0.293
	Age groups (years)			0.552		
	[12–20]	2/6	33.3 (4.3–77.72)		1 (reference)	
	[21–40]	5/27	18.5 (6.3–38.08)		0.379 (0.067–2.13)	0.271
	> 40	1/2	50 (1.2–98.74)		3.714 (0.205–67.14)	0.507
	Regions			0.156		
	Center	7/33	21.2 (8.9–38.90)		1 (reference)	
	Littoral	1/1	100 (2.5–100)		–	–
2013	Gender			0.633		
	Female	4/20	20 (5.7–43.66)		1 (reference)	
	Male	8/31	25.8 (11.85–44.61)		0.767 (0.196–3.003)	0.703
	Age groups (years)			0.250		
	[12–20]	2/7	28.6 (3.66–70.95)		1 (reference)	
	[21–40]	9/42	21.4 (10.29–36.81)		0.563 (0.117–2.706)	0.761
	> 40	1/1	100 (2.5–100)		–	–
	Regions					
	Center	12/50	24 (13.06–38.16)	0.575	–	–
2014	Gender			0.103		
	Female	1/33	3 (0.777–15.75)		1 (reference)	
	Male	6/43	14 (5.29–27.93)		0.193 (0.02–1.686)	0.137
	Age groups (years)			0.777		
	[12–20]	3/20	15 (3.2–37.89)		1 (reference)	
	[21–40]	4/52	7.7 (2.1–18.53)		0.583 (0.12–2.83)	0.452
	Regions			0.013		
	Center	5/67	7.5 (2.4–16.56)		1 (reference)	
	South	2/3	66.7 (9.4–99.15)		27.20 (2.089–354.15)	0.012
2015	Gender			0.103		
	Female	3/39	7.7 (1.6–20.87)		1 (reference)	
	Male	10/50	20 (10.03–33.71)		0.33 (0.085–1.307)	0.115
	Age groups (years)			0.239		
	< 12	1/1	100 (2.5–100)		1 (reference)	
	[12–20]	3/25	12 (2.5–31.21)		0.917 (0.255–3.290)	0.894
	[21–40]	8/61	13.1 (5.8–24.21)		0.694 (0.205–2.35)	0.431
	> 40	1/2	50 (1.2–98.74)		6.25 (0.366–106.76)	0.206
	Regions			0.039		
	Center	11/78	14.1 (7.2–23.83)		1 (reference)	
	East	1/1	100 (2.5–100)		–	–
	North west	1/1	100 (2.5–100)		–	–
2016	Gender			0.477		
	Female	7/47	14.9 (6.2–28.30)		1 (reference)	
	Male	7/67	10.4 (4.3–20.34)		1.55 (0.487–4.604)	0.479
	Age groups (years)			0.830		
	[12–20]	5/29	17.2 (5.8–35.77)		1 (reference)	
	[21–40]	8/81	9.9 (4.3–18.5)		0.493 (0.157–1.55)	0.227
	> 40	1/1	100 (2.5–100)		–	–
	Regions			0.029		
	Center	11/98	11.2 (5.7–19.19)		1 (reference)	
	East	2/4	50 (6.7–93.24)		8.167 (1.052–63.41)	0.045
	South	1/1	100 (2.5–100)		–	–
2017	Gender			0.120		
	Female	3/22	9.4 (2.9–34.91)		1 (reference)	
	Male	7/29	24.1 (10.29–43.54)		0.325 (0.075–1.402)	0.132
	Age groups (years)			0.723		
	[12–20]	3/21	14.3 (3.04–36.34)		1 (reference)	
	[21–40]	7/38	18.4 (7.7–34.32)		0.664 (0.154–2.874)	0.584
	Regions			0.114		
	Center	8/51	15.7 (7.02–28.58)		1 (reference)	
	East	1/5	20 (0.5–71.64)		1.306 (0.130–13.08)	0.821
	Littoral	1/1	100 (2.5–100)		–	–

^a^Information from available data

^b^ number of tested patients by categories may not sum to total because of missing data; %, percentage of anti-HDV prevalence among HBsAg positive patients; +, positive; N, total

p^c^, estimated by Chi-squared of Pearson test; p, it is the p value associated with the OR; OR, odds ratio; CI, confidence interval

### Discussion

Our results confirm that Cameroon is still a highly endemic country for HDV infection as reported by previous studies which showed prevalence’s between 6.5 and 27.3% among HBsAg carriers [7–12].

A recent study conducted in Cameroon based on analysis of 1928 HBsAg positive specimens from retrospective viral surveillance studies during the period 2010–2016 also confirmed a high seroprevalence of HDV at 46.73% [13]. HDV seroprevalence reported in Cameroon could vary with the study population and assays used for screening.

Although there was no significant difference in anti-HDV prevalence with respect to the year of sampling in our study, an important decrease was observed in 2014. This could be explained by the fact that people older than 40 years who were found to be the most affected were less represented during this year. In 2015, 2016 and 2017, the anti-HDV prevalence remained constant. We also notified that people less than 12 years old were apparently less infected; this result can be explained with the fact that they were born at the moment the anti-HBV vaccine was introduced in the expanded program on immunization in Cameroon in 2005. Our data highlight the potential impact of HBV vaccination on the reduction of HDV burden in Cameroon. The East and South regions of Cameroon were the most affected by hepatitis delta infection, probably because these regions are located in Cameroon’s dense forest. The highest prevalence’s of delta infection worldwide have been reported in Amazonian forests [14]. Our results are consistent with previous findings [10] which also found that the most affected region of Cameroon was the East region. However, this observation was reflected in only a few years when performing year-specific analysis probably due to the small size of this population. Further studies are therefore needed to better understand the high prevalence of hepatitis delta infection in forest zones compared to other geographical areas. This study also highlighted a significant association between people over 40 years old and the hepatitis delta infection. Our results are consistent with some previous studies in Mauritania in which people greater than 33 years old were the most affected population; 42% among blood donors and 33.3% among pregnant women respectively [15, 16]. This might be due to nosocomial transmission during the early twentieth century as suggested by Pépin et al. in a study conducted in Cameroon [17]. Indeed, medical interventions including intravenous antimalarial drugs and transfusions have been found to have contributed to the transmission of hepatitis C virus and other blood borne viruses in an older population of ≥ 60 years [16]. Indepth studies are also required to better elucidate this observation. The major limits of our study was the lack of a representative population size per category and the fact that hepatitis D RNA levels were not checked. Despite these limitations, our study provides information about the evolutionary trend of hepatitis Delta virus.

### Conclusion

We conclude that Cameroon remains an endemic country for HDV infection with an average prevalence of 16.5%. Older age (> 40 years) and living in close proximity to the dense forest may be risk factors for Hepatitis D infection.

## Limitations

The limitation of this study was the few socio-demographic factors available from the CPC database, which limits robustness of the study.

## Data Availability

All data generated or analyzed during this study are available from RN.

## Abbreviations

HDV: hepatitis D virus
HBsAg: hepatitis B surface antigen
CPC: Centre Pasteur of Cameroon
ELISA: enzyme linked immunosorbent assay
